# UPEC kidney infection triggers neuro-immune communication leading to modulation of local renal inflammation by splenic IFNγ

**DOI:** 10.1371/journal.ppat.1009553

**Published:** 2021-05-20

**Authors:** Svava E. Steiner, Ferdinand X. Choong, Haris Antypas, Carlos E. Morado-Urbina, Anette Schulz, Alex Bersellini Farinotti, Duygu B. Bas, Camilla I. Svensson, Agneta Richter-Dahlfors, Keira Melican

**Affiliations:** 1 AIMES—Center for the Advancement of Integrated Medical and Engineering Sciences, Karolinska Institutet and KTH Royal Institute of Technology, Stockholm, Sweden; 2 Department of Neuroscience, Karolinska Institutet, Stockholm, Sweden; 3 Department for Physiology and Pharmacology, Center for Molecular Medicine, Karolinska Institutet, Stockholm, Sweden; University of California, Davis, UNITED STATES

## Abstract

Bacterial infection results in a veritable cascade of host responses, both local and systemic. To study the initial stages of host-pathogen interaction in living tissue we use spatially-temporally controlled *in vivo* models. Using this approach, we show here that within 4 h of a uropathogenic *Escherichia coli* (UPEC) infection in the kidney, an IFNγ response is triggered in the spleen. This rapid infection-mediated inter-organ communication was found to be transmitted via nerve signalling. Bacterial expression of the toxin α-hemolysin directly and indirectly activated sensory neurons, which were identified in the basement membrane of renal tubules. Nerve activation was transmitted via the splenic nerve, inducing upregulation of IFNγ in the marginal zones of the spleen that led to increasing concentrations of IFNγ in the circulation. We found that IFNγ modulated the inflammatory signalling generated by renal epithelia cells in response to UPEC infection. This demonstrates a new concept in the host response to kidney infection; the role of nerves in sensing infection and rapidly triggering a systemic response which can modulate inflammation at the site of infection. The interplay between the nervous and immune systems is an exciting, developing field with the appealing prospect of non-pharmaceutical interventions. Our study identifies an important role for systemic neuro-immune communication in modulating inflammation during the very first hours of a local bacterial infection *in vivo*.

## Author summary

Our understanding of how the host responds to bacterial infection is constantly evolving. Here we use complex animal and cell culture models to demonstrate how nerves can sense a bacterial infection in the kidney and transmit that information to the spleen within a few hours. We show that the bacterial toxin α-hemolysin is a trigger for the nervous reflex which leads to increased IFNγ production in the spleen, which can be detected in the blood. IFNγ, in turn, modulates how the kidney cells respond to bacterial infection. Our work identifies a new role for nerves in sensing a local bacterial infection and rapidly activating a modulating systemic immune response within the very first hours of infection. Nerve driven immunity during bacterial infection is an expanding field. Here we contribute by highlighting a potential for nerves in monitoring and modulating the immune response during the early hour of infection. Understanding all interplaying factors of infection is crucial as we search for alternative targets for diagnosis or treatment of infection in the face of a post-antibiotic era.

## Introduction

A local bacterial infection triggers rapid physiological changes at the site of infection and in doing so alerts the systemic host defence. The innate immune response, which activates phagocytic cells, is often considered the first line of defence. It has become increasingly clear however that there are numerous responses that occur immediately upon bacterial infection, prior to inflammatory cell infiltration [1,2]. To study these very early infection time-points *in vivo*, we have developed highly refined models with exceptional spatial and temporal control, an approach we term *Tissue Microbiology* [3–5]. In the model used here, we microinfuse uropathogenic *Escherichia coli* (UPEC) directly into a single proximal tubule in an exteriorized rat kidney, allowing us to define the exact dose, timeframe and location of infection in living tissue. Using this method, we previously identified a number of pathophysiological responses that occur within the first minutes to hours of kidney infection, including activation of local vascular coagulation [2,6,7]. Combining our model with tissue transcriptomics, we showed that within 8 h of UPEC infection there was an overrepresentation of interferon-γ (IFNγ) regulated genes expressed at the kidney infection site. Unexpectedly, the IFNγ we detected in the serum of animals with kidney infection appeared to originate from the spleen, implying some type of inter-organ communication [8]. Several forms of communication networks have been described, including the traditional humoral responses *i*.*e*. cytokine signalling [9], and more recently a reflex-like nervous signalling [10,11].

Neuro-immune communication is an expanding area of research studying the interconnection between the immune and nervous systems [10,12,13]. The nervous system can influence inflammatory responses, receiving information from peripheral sites of inflammation via sensory neurons [14–17]. A major advantage of neuro-immune signalling is that the response kinetics of neurons is orders of magnitudes faster than humoral immunity [18,19]. Bacteria and bacterial products can activate sensory neurons and modulate pain and local inflammation [20–24], but the functional outcomes of bacteria nerve interaction in terms of the systemic host defense has not yet been described.

In this work, we applied our spatially-temporally controlled *in vivo* model to study the early systemic response to local kidney infection. We show that neuro-immune signalling communicates infection status between organs within the first 4 h. The spleen responds by upregulating IFNγ. IFNγ can then modulate inflammation in infected renal epithelial cells, demonstrating a role for nerves in the early coordinated host response to kidney infection.

## Results

### A localised UPEC infection in the kidney rapidly triggers IFNγ expression in the spleen

**Fig 1:**
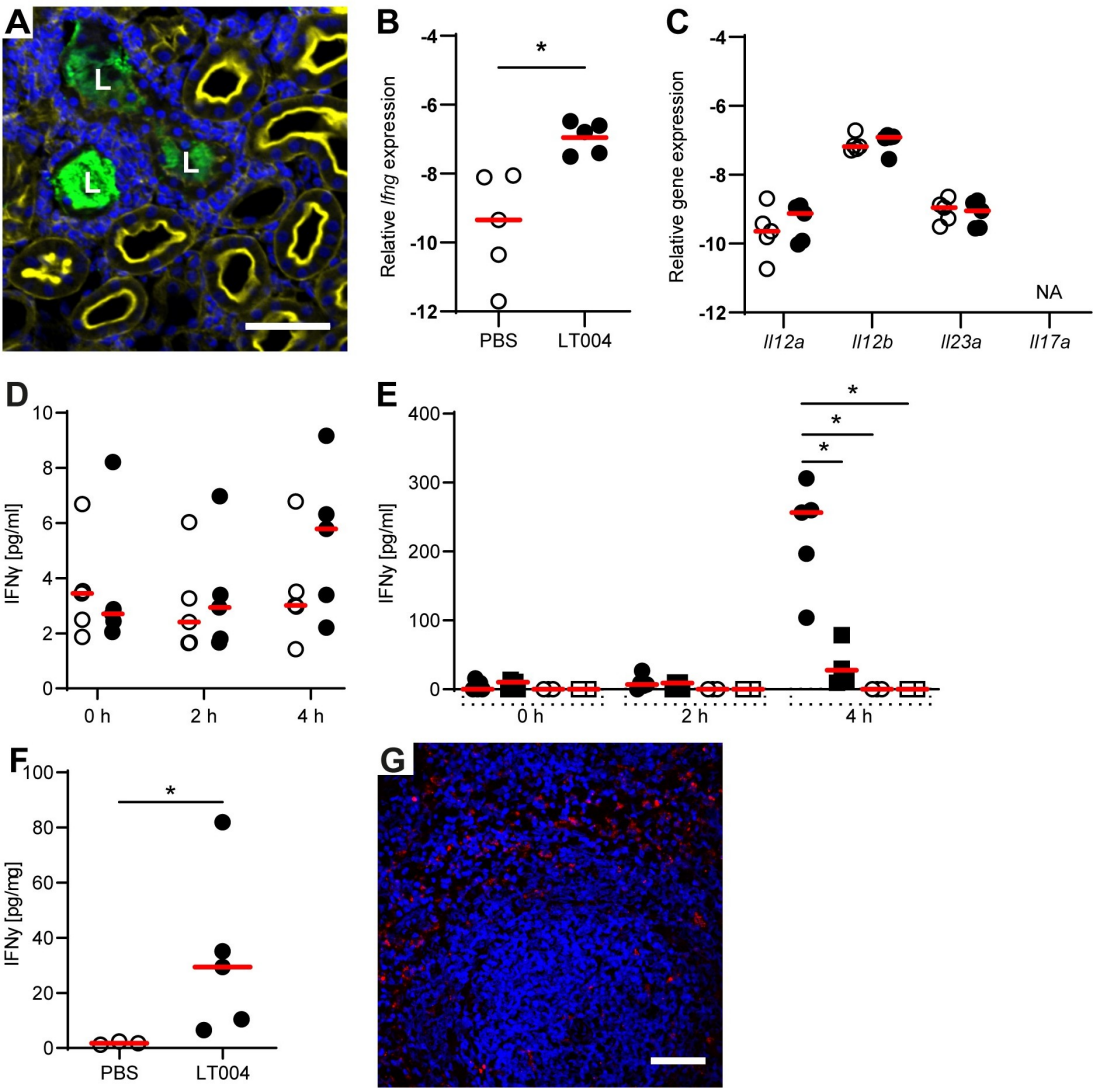
Local kidney infection triggers IFNγ expression in the spleen. (**A**) *Ex vivo* confocal imaging of kidney tissue 4 h after microinfusion of LT004 shows bacteria (green) localized to the lumen (L) of an infected tubule. Hoechst stain (blue) shows nucleated cells around the infected tubule. Actin staining (yellow) show the apical microvilli of uninfected proximal tubules. Scale bar = 50 μm. (**B**) Relative *Ifng* mRNA expression in spleens 4 h after kidney infection with LT004 (black circles) or sham-infection (PBS-infused, unfilled circles). Expression in relation to the reference gene *Gapdh* (delta CT) is shown.* = p< 0.05 determined by Kruskal-Wallis and Dunn’s correction (this group was run in parallel to the group shown in Fig 3D and therefore grouped analysis comparing three groups was performed). n = 5 in each group. (**C**) Relative *Il12a*, *Il12b*, *Il23* and *Il17* mRNA expression compared to the reference gene *Gapdh* (delta CT) is shown for LT004-infected (black circles) and sham-infected (PBS-infused, white circles) animals. NA = not amplified. (**D**) IFNγ in serum from rats with kidney infection (LT004, black circles) and sham-infected (PBS-infused, white circles) at 0, 2, and 4 h after microinfusion into one renal tubule. (**E**) IFNγ in serum from animals after sham-splenectomy (circles) or full splenectomy (squares). Black shapes indicate infection with LT004 (microinfusion into three renal tubules); white shapes represent PBS infusion (white). (**F**) ELISA measurement of IFNγ in splenic tissue from sham-splenectomised animals after 4 h of kidney infection with LT004 (black circles) or PBS-infusion (white circles). (**G**) *Ex vivo* confocal imaging of spleen of a sham-splenectomised animal 4 h after kidney infection with LT004 shows IFNγ (red) mainly in the red pulp and marginal zone, distinguished by nucleated cells (Hoechst stain, blue), scale bar = 50 μm. Individual data points and median values (red bars) are plotted in (B-F), n = 3–5 in each group. * = p< 0.05 determined by two-way ANOVA with Bonferroni’s or Turkey’s test in (D and E) respectively or Mann-Whitney in (C and F). Statistical significance was set to p<0.001 in (C) to adjust for multiple comparisons. Images in (A) and (G) are representative of n = 5.

Initially, we set-out to define the mechanisms of the inter-organ communication which had been implied in our previous transcriptomic work at 8 h post kidney infection [8]. We began by focussing on the rapidity of the response; how early after kidney infection could the spleen possibly react? Taking advantage of the spatial and temporal control of our infection model, we studied the splenic response just 4 h after kidney infection. Kidney infection was initiated by micro-infusing LT004, a GFP^+^ expressing variant of UPEC CFT073, into the lumen of a single renal proximal tubule. Microscopy analysis at 4 h showed a highly localised infection site with no visual indications of bacterial dissemination (**Fig 1A**), corroborated by negative blood cultures (**S1A Fig**). We then screened for splenic expression of *Ifng* after 4 h of kidney infection. A significant increase in IFNγ mRNA was found in the spleens of UPEC LT004 infected animals, compared to animals infused with PBS (sham-infection) (**Fig 1B**). To assess if the splenic IFNγ expression was being caused by a splenic inflammation (splenitis), we probed for IFNγ inducing IL-12 and IL-23 mRNA in the spleen but found no upregulation (**Fig 1C**). We also did not find splenic mRNA expression of IL-17a, a downstream cytokine of IL-23 (**Fig 1C**). Histological analysis did not show any visual splenitis in any animals (**S1B and S1C Fig**) implying that splenic inflammation was not directly responsible for IFNγ upregulation. Further, we screened serum for IL-12b/p40, a subunit of the IFNγ inducing cytokines IL-12 and IL-23, but did not find any (**S1D Fig**). Together this suggested that the induction of splenic IFNγ in response to kidney infection was not being mediated by the typical cytokine inflammatory pathways.

To evaluate if the splenic upregulation of *Ifng* at 4 h translated to a systemic response, as we had previously reported at 8 h [8], we screened serum. After 4 h we could detect the first signs of increasing amounts of IFNγ following the infection of a single renal tubule (**Fig 1D**). Our next aim was to define if the spleen was the primary contributor to the detected serum IFNγ by performing a complete splenectomy prior to kidney infection. As microinfusion into just one renal tubule caused increasing, but not significantly elevated levels of IFNγ (**Fig 1D**), we wanted to induce a more robust IFNγ serum response to facilitate differentiation. This was achieved by microinfusing bacteria into 3 renal tubules in each animal. Intravital multiphoton imaging confirmed the localisation of the infusions (**S1E and S1F Fig**). We were able to detect significantly increased serum IFNγ levels in infected, sham-splenectomised animals (animals who underwent preparation for splenectomy but the spleen was not removed) (**Fig 1E**), confirming that infection in 3 tubules did indeed result in significant IFNγ serum protein after 4 h of kidney infection. Animals who underwent full splenectomy prior to infection however demonstrated significantly lower serum IFNγ (**Fig 1E**), as well as increased bacterial dissemination (**S1G Fig**). Further confirming that the spleen was producing IFNγ following kidney infection, ELISA analysis of spleens from infected, sham-splenectomised animals displayed significantly higher IFNγ levels compared to those infused with PBS. (**Fig 1F**). To investigate which areas in the spleen produced IFNγ during kidney infection, we performed immunohistological analysis on spleens from sham-splenectomised animals and identified IFNγ predominantly in the red pulp and marginal zones of infected, but not PBS infused animals (**Figs 1G and S1H**). Together this data revealed that the spleen produces IFNγ within 4 h of a local kidney infection, and that it is the major contributor to the observed systemic IFNγ.

### Signalling via the splenic nerve is required for inter-organ communication of infection

The speed of this observed inter-organ communication raised the question: how does the spleen know so quickly that the kidney is infected? Our data above, combined with the extremely early timeframe, implied that the inter-organ communication triggered by kidney infection may not be driven by typical humoral inflammatory pathways, and sparked our interest in other signalling pathways.

**Fig 2:**
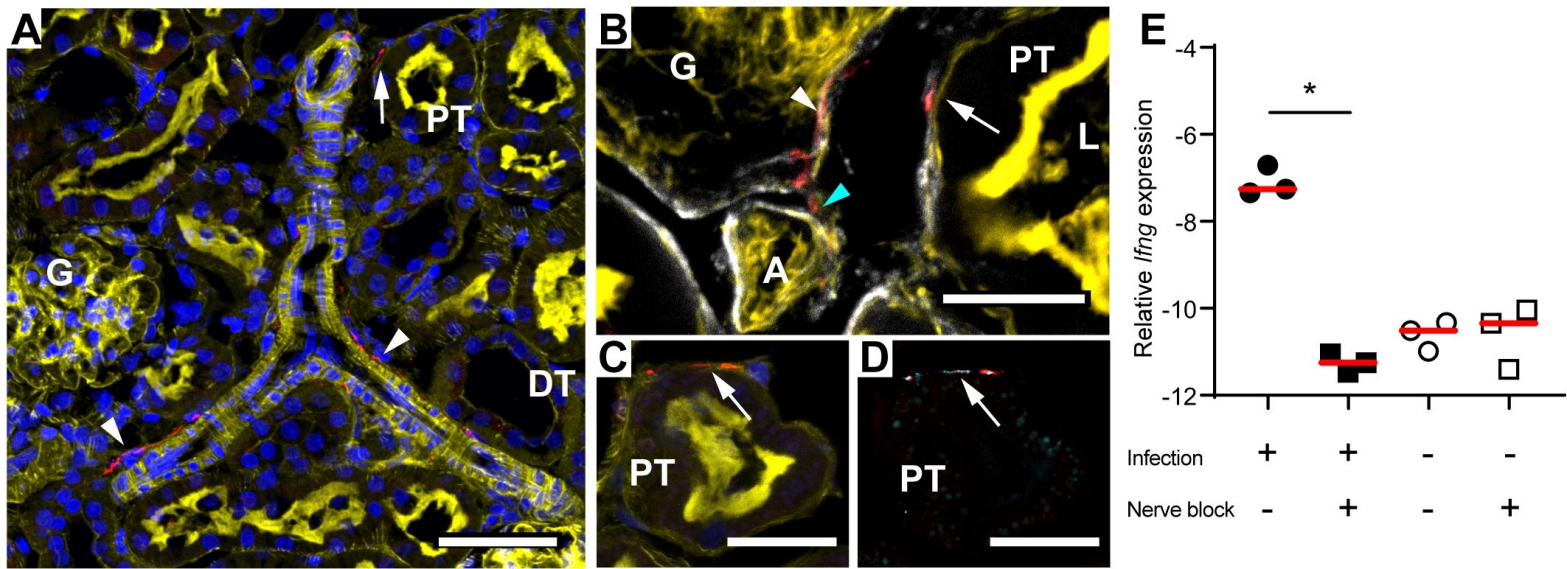
Nervous signalling is required for inter-organ communication. (**A-D**) *Ex vivo* confocal microscopy of rat kidney. (**A**) Actin (yellow) and Hoechst stain (blue) show proximal tubules (PT), distal tubules (DT), and glomerulus (G). β3-tubulin (red) shows nerve fibres surrounding the vasculature (arrow heads) as well as at the basal face (arrow) of proximal tubules. Scale bar = 50 μm. (**B**) Nerve fibres stained by β3-tubulin (red) co-localize with collagen IV-rich (white) basement membrane of the proximal tubule (arrow), glomeruli (white arrow head), and arteriole (A) (cyan arrow head). L = lumen of proximal tubule. Scale bar = 15 μm. (**C-D**) Co-staining with **(C)** the general marker of nerve fibres anti-PGP9.5 (red) and (**D**) the sensory nerve marker anti-TrkA (cyan) locates sensory nerve fibres at the basal face of actin (yellow) rich proximal tubules (arrows). Scale bar = 20 μm. Images are representative of n = 3. (**E**) mRNA expression of *Ifng* in splenic tissue after 4 h kidney infection with LT004 (black symbols) or sham-infection (PBS-infused, unfilled symbols), with (squares) or without (circles) nerve block prior to infection. Relative *Ifng* expression (delta CT) is given in relation to the reference gene Gapdh. Individual data points and median values (red bars) are shown. * = p<0.05 determined by Kruskal-Wallis analysis and Dunn’s correction, n = 3 for each group.

To explore if a neuro-immune pathway may act as the sensing/signalling mechanism in our model, we stained for sensory nerves in the kidney. β3-tubulin positive nerve fibres were found surrounding the intra-renal vasculature and tubules in the kidney cortex (**Fig 2A**). Co-staining with collagen IV identified neural projections in the basement membrane of proximal tubules, glomerulus and arterioles (**Fig 2B)**. Co-staining of the general nerve marker PGP9.5 and TrkA, a marker for sensory nerves, revealed sensory nerves located at the basal face of renal proximal tubules (**Fig 2C and 2D**). This highlighted the potential for nerve driven communication during kidney tubule infection. To test whether nerve conduction was essential for the splenic response, we placed a pharmacological nerve block locally on the splenic nerve prior to kidney infection. Blocking nerve conduction of the splenic nerve completely abrogated the increase in splenic *Ifng* expression following kidney infection (**Fig 2E**). Controls confirmed that a sham nerve block did not affect splenic *Ifng* expression, and the nerve block itself did not induce upregulation of *Ifng*. This data demonstrated that intact nerve conduction to the spleen is required for inter-organ communication following kidney infection, directly implicating neuro-immune signalling as a key mediator of this response.

### Neuronal signalling is triggered directly and indirectly by the UPEC toxin HlyA

**Fig 3:**
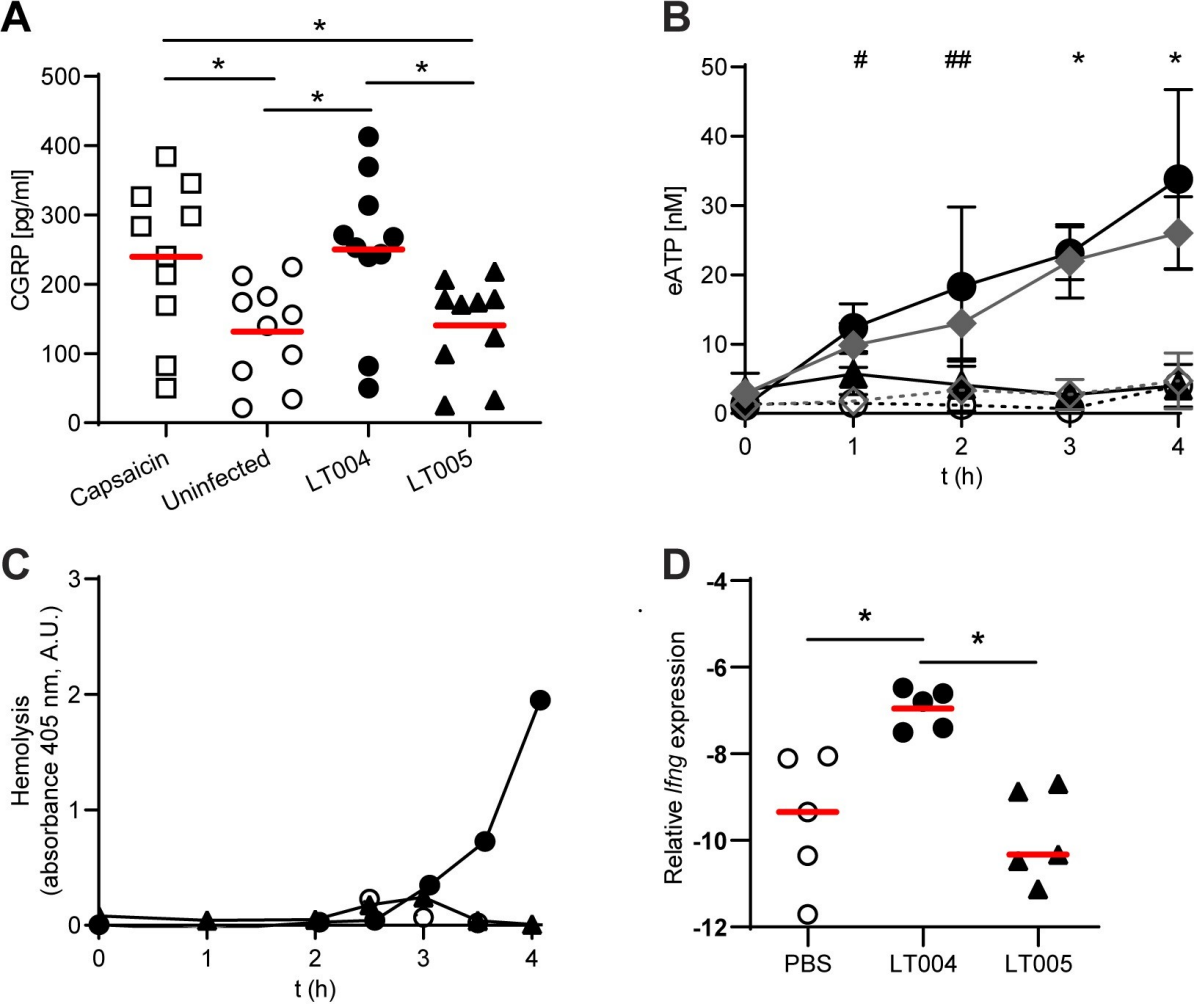
A role for HlyA in neuronal signalling during UPEC kidney infection. (**A**) Stimulation of primary DRG cells with LT004 (HlyA+, black circles) and LT005 (HlyA-, black triangles) for 4 h, with capsaicin (unfilled squares) as positive control and uninfected cells (unfilled circles) as negative control. Individual values and means (red bar) are shown, n = 10. * = p<0.05 calculated by one-way ANOVA with Turkey’s correction for multiple comparisons. (**B**) eATP levels in flow-through media from renal epithelial A498 cells infected with CFT073 (HlyA+, black circles), LT002 (HlyA-, black triangles) and ARD371 (LT002 pBAD-HlyA+, grey diamonds), or uninfected (unfilled circles) and uninfected cells exposed to 0.2% arabinose (unfilled diamonds) under flow. Graph shows means ± SD, n = 3 for each condition. # = p<0.05 of CFT073-infected compared to uninfected cells, ## = p<0.05 of CFT073-infected compared to LT002-infected and uninfected cells, and * = p<0.05 of CFT073- and ARD371-infected compared to LT002-infected and uninfected cells, calculated by two-way ANOVA and Turkey´s correction for multiple comparisons. (**C**) Hemolytic activity, measured as arbitrary units (A.U.), in the flow-through media from cells infected with CFT073 (HlyA+, black circles), LT002 (HlyA-, black triangles) or uninfected (unfilled circles). Representative of n = 3, see Figure S2 for repeats. (**D**) Relative *Ifng* mRNA expression in spleens 4 h after kidney infection with LT005 (black triangles). Data for infection with LT004 (black circles) and sham-infection (PBS-infused, unfilled circles) is re-visualized from Fig 1B for comparison. Expression in relation to the reference gene *Gapdh* (delta CT) is shown. * = p< 0.05 determined by Kruskal-Wallis and Dunn’s correction. n = 5 in each group.

We next aimed to determine the molecular mechanisms by which bacterial kidney infection could activate local sensory nerves. To do this we isolated primary dorsal root ganglia (DRG) cells, which are sensory pseudounipolar nerve cells. Directly infecting DRG cells with the UPEC strain LT004 led to a significantly increased release of CGRP, indicative of a neuronal response (**Fig 3A**). Our previous work had shown that the UPEC exotoxin α-hemolysin (HlyA) is expressed *in vivo* and influences the early kinetics of kidney infection in our model [2]. Alongside reports that bacterial toxins could trigger nerve responses [20], we evaluated the role of HlyA in this signalling. We infected DRG cells with LT005, an isogenic mutant of LT004, which lacks expression of HlyA, and found no increase in supernatant CGRP levels (**Fig 3A**). To confirm the role of HlyA, we complemented LT005 with plasmid born HlyA expression (pBAD-HlyA) under the control of an arabinose inducible promoter, creating strain ARD372 (**S1 Table**). Hemolysis was restored when ARD372 was grown in 0.2% arabinose (**S2A Fig**). Infection of DRG cells with ARD372 showed a significantly increased CGRP response (**S2B Fig**). These results implicate expression of the UPEC HlyA toxin as an important factor in stimulating a direct neuronal response in nerve cells.

While some bacteria may directly come into contact with the tubule basement membrane and thereby nerves during infection with LT004 *in vivo* (**S2C Fig**), typically the bacteria encounter kidney epithelial cells first. We therefore wanted to understand the role of kidney epithelial cells in the signalling of infection to the neurons. Reports have shown that neuroactive eATP is among the first molecules released by infected epithelial cells in other models [25,26], and we confirmed that exposure of primary DRG cells to eATP triggered a robust CGRP response (**S2D Fig**). We then tested whether UPEC bacteria could induce eATP release from kidney epithelial cells. To simulate the renal tubular microenvironment, we used a biomimetic microfluidic model system. Renal epithelial cells (A498) grown in microfluidic chambers were infected with UPEC CFT073 (HlyA+) under physiological flow (75 μl/min). The CFT073 strain, which does not express GFP+ was used in these experiments to avoid interference of our experimental read-out. Epithelial eATP secretion, detected in the flow-through media, increased significantly within 1 h of CFT073 infection, with eATP levels continuing to increase up to 4 h (**Fig 3B**). Bacteria alone did not secrete any detectable eATP (**S2E Fig**). We then tested the epithelial response to LT002, an isogenic strain of CFT073 with a mutation in *hlyA*, and found that a lack of HlyA abrogated epithelial eATP secretion (**Fig 3B)**. Complementing LT002 with pBAD-HlyA (creating ARD371) recovered hemolysis (**S1 Table and S2A Fig**). Infection of A498 cells with ARD371 restored the eATP response to similar levels as the CFT073 strain **(Fig 3B**). This data indicates that bacterial expression of HlyA is critical to the epithelial eATP response at these early time points.

Previously our lab has shown that HlyA exerts a concentration dependent, pro-inflammatory action on target cells [27,28]. To study how this biphasic action may modulate epithelial eATP secretion, we analysed the level of hemolytic activity in flow-through media. No hemolysis was detected in flow-through media from cells infected with LT002 (HlyA-) or uninfected controls, whereas a sharp rise was observed at 3–4 h in CFT073 (HlyA+) infected cells (**Figs 3C and S2F–S2G**). Increasing hemolysis was also observed over time in flow through of cells infected with the HlyA complemented strain ARD371 (**S2H–S2J Fig**). Comparing the kinetics of hemolysis to eATP release, our data indicated that eATP release occurred at sub-hemolytic concentrations of HlyA (**Figs 3B and 3C and S2F–S2J**). This result was validated by time-lapse microscopy of cell morphology during infection under flow. Cells infected with LT002 (HlyA-) remained adherent, with little change in morphology despite heavy bacterial infection (**S2K Fig and S1 Video**), while infection with CFT073 (HlyA+) caused cell rounding and detachment coinciding with the increasing hemolysin levels (**S2K Fig and S2 Video**). Annexin V staining showed induction of apoptosis in cells infected with CFT073 (HlyA+), but not LT002 (HlyA-), while the cells remained generally non-necrotic throughout the 4 h experiment (**S2L Fig)**. As HlyA appears to trigger apoptosis rather than necrosis, our collective results indicate that sub-lytic concentrations of HlyA acts as an inducer of neuroactive eATP release from UPEC-infected renal epithelial cells during the early time-points of infection.

To understand if the role we observe for HlyA *in vitro* is relevant *in vivo*, we used our *Tissue Microbiology* model. LT005, the isogenic mutant of LT004 lacking expression of HlyA (HlyA-) was infused into a kidney tubule as described above and the splenic expression of *Ifng* measured after 4 h. While pathophysiological changes similar to that of infection with LT004 (HlyA+) were observed (**S2M Fig**), infection with LT005 did not cause any upregulation of *Ifng* compared to PBS-infused animals (**Fig 3D**). In comparison to LT004 infused animals (Fig 1B and shown again for reference in 3D Fig), who showed a significant increase in splenic *Ifng*, this indicates that HlyA does indeed play an important role in early kidney-spleen communication *in vivo*. Interestingly, this work also downplayed the role of another key bacterial virulence factor, the endotoxin lipopolysaccharide (LPS). LT004 and LT005 have the same LPS structure and the lack of signalling from LT005 indicates that LPS is not a vital element in this early inter-organ communication. Confirming this, we performed immunohistological analysis and found that there was no LPS present in the splenic tissue of LT004 infected animals (**S2N Fig**).

Collectively, our findings show that HlyA expression is essential for triggering a nerve-driven inter-organ communication between kidney and spleen upon bacterial infection. This works adds an exciting new component to the multifaceted role HlyA plays during kidney infection.

### IFNγ modulates pro-inflammatory signalling in UPEC infected kidney epithelia

**Fig 4:**
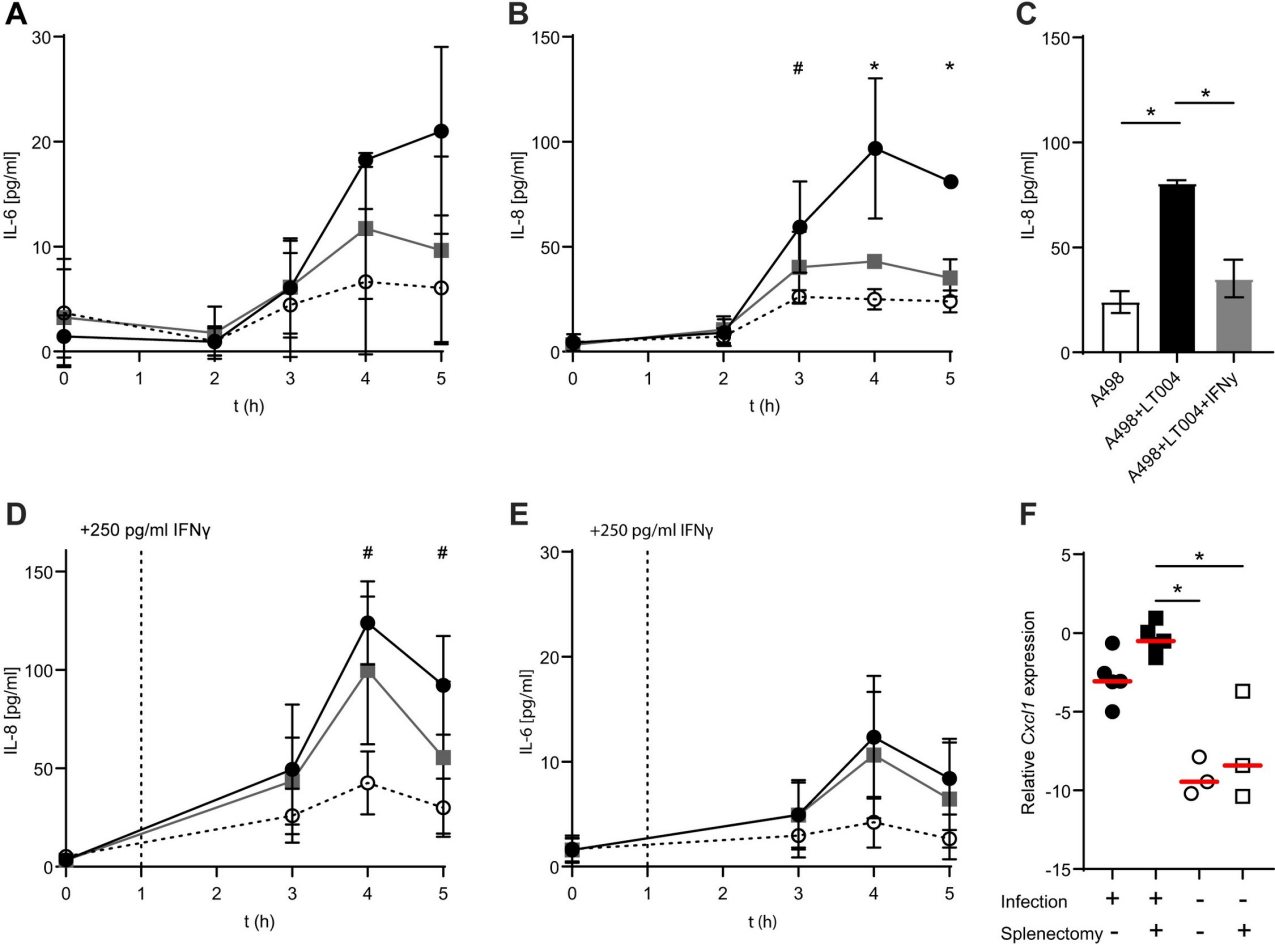
IFNγ dampens proinflammatory signalling from infected kidney cells. (**A-B**) ELISA measurements of (**A**) IL-6 and (**B**) IL-8 release over time from renal epithelial A498 cells under flow, infected with LT004 (black circles), no infection (unfilled circles) or infection with addition of 250 pg/ml IFNγ (grey boxes), n = 3. The IL-8 data from the 5 h timepoint is re-visualized in (**C**) to show statistical significance. ELISA measurement of (**D**) IL-8 and (**E**) IL-6 release over time from A498 cells infected with LT004 under flow (black circles), uninfected (unfilled circles) or infected with the addition of 250 pg/ml IFNγ 1 h post infection (grey boxes), n = 4. Graphs in A-E show means ± SD. Significance was calculated by two-way ANOVA, with Turkey’s correction for multiple comparisons. * = p<0.05 with significance for comparison between uninfected and both LT004 infected and LT004 infected cells co-incubated with 250 pg/ml IFNγ. # = p<0.05 with significance for comparison between uninfected and LT004 infected cells. (**F**) *Cxcl1* mRNA expression in sham-splenectomised (circles) or splenectomised (squares) animals, either infected (black symbols) or PBS-infused (unfilled symbols). Gene expression is given in relation to the reference gene *Gapdh* (delta CT). Individual data points and median (red bar) are shown. n = 3–5 in each group. * = p<0.05 calculated by Kruskal-Wallis analysis and Dunn’s correction.

While we had identified HlyA as a bacterial factor triggering nerve-driven communication between the kidney and the spleen, the question remained, what is the role of splenic IFNγ in local kidney infection? IFNγ is commonly considered a pro-inflammatory cytokine, but it has also been found to have anti-inflammatory effects on both immune and non-immune cells [29–33]. To address the possible effects of IFNγ on inflammatory signalling *in vivo*, we micro-dissected out the infection sites from animals with and without splenectomies and performed Luminex screening. We were, however, unable to identify significant differences in protein cytokine levels (**S3A–S3G Fig**). We believe this is likely due to a lack of sensitivity of these methods at this very early infection time point. Supporting this, we analysed MPO activity, an indicator of neutrophil recruitment, in the dissected tissue and found no apparent differences in MPO activity (**S3H Fig**). This demonstrated a lack of discernible inflammation at 4 h post infection with tissue analysis methods. We therefore focused on studying the role of IFNγ on renal epithelial cells, the main infected cell type at 4 h. We began by defining how renal epithelial cells (A498) responded to infection. Using the biomimetic flow-chamber approach, we infected A498 renal epithelial cells under flow with the UPEC strain LT004, and screened the flow-through media by Luminex screening. Over the 4 h of infection we detected increasing amounts of IL-8 (**S4A Fig**) and IL-6 (**S4B Fig**). To confirm this, we repeated the experiments and analysed IL-8 and IL-6 using ELISA, and found significantly increasing levels of IL-8 over time, but not IL-6 (**Fig 4A–4C**). No increases of other tested cytokines (including IL-1ß, TNFα, IL-12, IL-18, IFNγ and IL-10) were found (**S4C–S4H Fig**). The lack of direct IFNγ release from the infected renal epithelial cells (**S4G Fig**) and the absence of IFNγ in infected renal tissue (**S3F Fig**), further highlighted the spleen as the major producer of IFNγ during kidney infection.

To test how IFNγ may modulate the inflammatory IL-8 and IL-6 responses from the epithelial cells, we added IFNγ into the medium of the biomimetic flow-chamber one hour before infection and measured the inflammatory response. Addition of IFNγ had no effect on bacterial viability (**S4I Fig**) or renal epithelial cell morphology (**S4J Fig**). While IFNγ had little effect on the IL-6 response (**Fig 4A**), 250 pg/ml of IFNγ effectively reduced the IL-8 response to the level of uninfected cells (**Fig 4B,** and the 5 h time-point re-visualized in **4C**). This data implied a role for IFNγ in modulating the renal epithelial inflammatory response to UPEC infection. To model the proposed *in vivo* kinetics more closely, we allowed time for the epithelial infection to establish itself within the microfluidic system prior to the addition of IFNγ. Addition of IFNγ 1 h after establishment of infection still resulted in a dampening of the epithelial release of IL-8 (**Fig 4D**), but not IL-6 (**Fig 4E**). This data suggests a role for splenic IFNγ in modulating renal inflammation and specifically IL-8. Returning to our *in vivo* model, we investigated whether we could see this effect at an mRNA level at this very early time point. We found that animals who had undergone splenectomy had increased expression of *Cxcl1* (homologous to human IL-8) at the infected tissue site in the kidney, when compared to PBS-infused animals (**Fig 4F**). Animals who underwent only sham-splenectomy prior to infection did not show significantly higher levels than the PBS-infused controls (**Fig 4F**). This indicated that the absence of the spleen, and corresponding drop in serum IFNγ, results in higher levels of pro-inflammatory signalling in the kidney at 4 h post infection. In combination with our previous transcriptomic data, which showed a significant increase in a number of IFNγ regulated genes during kidney infection [8], this work highlights an important modulatory role of splenic IFNγ within the first hours of kidney infection.

## Discussion

The speed by which an infection can be identified, and how the defence mechanisms are triggered is critical to infection outcome. Here we show how the nervous system contributes to detecting bacterial kidney infection, communicates with the spleen within the first hours of infection and results in a modulating inflammatory response at the kidney infection site. Our complex model systems were critical to reveal the role of nerve driven immunity at this exceedingly early time-frame, giving new insight into the first phase of host-pathogen interactions.

Bacterial protein toxins play a major role in host-pathogen interactions. Our work here demonstrates a new role for the UPEC HlyA toxin in mediating a previously undescribed neuronal signalling during kidney infection. It was work from our lab a number of years ago which first described a biphasic pro-inflammatory action of the UPEC toxin HlyA which prompted re-evaluation of HlyA as a purely cytolytic toxin [27]. Subsequent to that finding, we and others have shown that HlyA influences a number of aspects of host-pathogen interaction during UTI including modulation of the host vascular response [2,34] and disruption of cell adhesion and inflammatory pathways [35–37]. Our current work adds a further component to HlyA activity, demonstrating that HlyA can stimulate the neuro-immune axis to trigger a systemic immunomodulatory response. Previously, *Staphylococcus aureus* α-toxin and the *Streptococcus pyogenes* Streptolysin S (SLS) toxin have been shown to directly activate sensory neurons, causing pain and modulation of local inflammation [20–22]. Our work extends these findings by demonstrating how toxin-nerve interaction rapidly triggers a systemic inter-organ communication of the kidney infection status to the spleen. We show two ways in which HlyA may trigger the nerve response, directly by stimulation of sensory neurons, as well as indirectly via eATP release from infected renal epithelial cells. These findings extend earlier work which describes eATP as a key regulator in mucosal inflammation the early phases of bacterial infection [25,26].

IFNγ is commonly considered a pro-inflammatory cytokine [30], and is known to sensitize tissue resident macrophages, as well as circulating monocytes [38–40]. During kidney infection, IFNγ stimulation has been shown to increase TLR4 and TLR2 expression in infected kidneys [41]. This suggests that IFNγ has a potential to enhance immunosurveillance and the response to bacteria during kidney infection. IFNγ has also been found to have anti-inflammatory effects, including an ability to downregulate IL-8 secretion in both immune [29,31] and non-immune cells [32,33]. Our data demonstrates a further seemingly anti-inflammatory role for splenic IFNγ in modulating IL-8 expression in kidney epithelial cells following infection. Ongoing research will be needed to evaluate the effect of the early-phase IFNγ response shown here on long-term infection outcomes.

Our results demonstrate an important role of the spleen in mediating a neuro-immune response to kidney infection. The spleen has been described as a control node for peripheral inflammation for a number of neuro-immune pathways [10]. The exciting finding of this neuro-immune communication has opened for non-pharmacological approaches addressing these neural reflex pathways [10,12,42]. Direct electrical stimulation of the vagus nerve prior to kidney ischemic-reperfusion injury was shown to significantly reduce the severity of injury and the systemic TNF levels [43]. During LPS mediated endotoxemia, vagus nerve stimulation has been shown to reduce TNF levels [44]. Our findings reveal an important role for nerve driven inter-organ communication during acute kidney infection. This will contribute to the ongoing developments in the field, highlighting the potential of nerve monitoring or modulation as an alternative target for diagnosis or adjunctive treatment of infection as we face a world in which antibiotics are becoming increasingly less effective.

## Materials and methods

### Ethics statement

All experimental procedures involving animals were conducted in accordance with guidelines established by Swedish and European regulations for the care and use of laboratory animals (Directive 2010/63/EU). All animal procedures were approved by the Stockholms Norra Djurförsöksetiska Nämnd (Sweden) with ethics permit numbers: N620/12, N35/14, N183/15, 10031/2017, 2-4038-2019 and 9013–2020. Procedural details are outlined below.

### Bacterial strains and growth conditions

Bacterial strains used in this study are listed and described in **S1 Table**. For all experiments UPEC was grown overnight in Luria-Bertani (LB) medium at 37°C, shaking, in the presence of kanamycin (Km, 50 μg/ml, Sigma-Aldrich, Sweden) or ampicillin (Amp, 100 ug/ml, Sigma-Aldrich, Sweden) when indicated. On the day of the experiment a fresh culture was cultivated to a density of OD_600_ = 0.5–0.6 (≈ 1–2 x 10^8^ CFU/mL). Bacteria were used directly for infection in the biomimetic model, but otherwise washed twice in PBS before use. ARD371 (LT002 pBAD-HlyA) and ARD372 (LT005 pBAD-HlyA) were constructed by cloning *hlyA* of CFT073 into the pBAD vector under the control of an arabinose-inducible promoter. Briefly, the open reading frame of *hlyA* was PCR amplified from the CFT073 gDNA using primers Sacl_hlyA_FW and BstBl_hlyA_RV (**S1 Table**) and subjected to double digestion (SacI-HF and BstBI enzymes, NEB) and purification (GE healthcare DNA purification kit), and was ligated to the pBAD plasmid, resulting in pBAD-HlyA. The pBAD-HlyA plasmid was electroporated into LT002 (CFT073 Δ*hlyA)* and LT005 (LT002 gfp+). Induction of *hlyA* was achieved by adding 0.2% arabinose to the media. All constructs were confirmed by genomic sequencing and the expression of hemolysin confirmed using a hemolytic assay (**S2A Fig**).

### Micro-perfusion procedures

Male Sprague-Dawley rats (245 ± 65 g) had free access to chow and water. Animals were housed with environmental enrichment and 12 h light/dark cycles. Rats were anaesthetized by intraperitoneal injection of 130–150 mg/kg thiobutabarbital sodium salt hydrate (Inactin hydrate, Sigma-Aldrich). Animals underwent a tracheotomy and cannulation of the femoral vein for infusion of 0.9% saline (1.5 ml/h) and removal of blood samples, and left ureter for urine sampling and to prevent bacteria from reaching the bladder. Core body temperature was monitored rectally and maintained using heating pads. Tubular micro-perfusion and induction of infection was performed as earlier described [2,6,7]. A fresh culture of bacteria was concentrated to 10^9^ CFU/ml in a PBS solution containing 1 mg/ml Fast Green FCF (Sigma-Aldrich) and 0.2 mg/ml cascade blue-conjugated 10 kDa dextran (ThermoFisher Scientific) or 0.2 mg/ml TRITC-conjugated 4 kDa dextran (TdB Labs). Bacterial suspensions or PBS control solution was loaded into pre-pulled glass micropipettes (5 μm tip size, World Precision Instruments). The left kidney was exposed via a subcostal flank incision, freed from surrounding fat, and supported in a kidney cup (Klaus Effenberger Med). Under stereoscopic microscope observation (100x), the bacterial suspension was infused over a period of 10 min into the lumen of superficial proximal tubules using a Leitz micromanipulator and a microinfusion pump, at a rate of 40 nl/min. At end point, animals were euthanised by infusion of KCl followed by exsanguination under full anaesthesia. Kidneys and spleen were removed for tissue analysis. CFU counts were obtained by plating samples on LB agar plates.

### Multiphoton microscopy

The kidney was externalized, stabilized in a kidney cup connected to the homeothermic table, and bathed in isotonic saline. A cover glass was secured on top of the kidney cup with vacuum grease. The infection sites were located using a 20X objective on the microscope (Multiphoton Galvometer on a *in vivo* Slicescope, Scientifica, UK). Excitation wavelength was 810 nm and laser power 20% (Mai Tai XF1-DS, Azpect, Sweden). Image stacks were collected by 1 μm optical steps to 100 μm depth.

### Splenectomy

Where indicated splenectomy was performed prior to kidney infection. The spleen was isolated from surrounding tissues and the vessels entering the spleen located. Vessels were cut between two ligatures (one proximal and one dorsal) to remove the spleen completely. In sham-splenectomised animals the vessels were isolated, but no ligatures were tied and no cuts made. After splenectomy the animals stabilized for 30 minutes, before continuing with infection.

### Blocking nerve conduction to the spleen

This protocol was adapted from previously described methods [43,45]. In indicated experiments, bupivacaine was applied locally to nerve fibres surrounding the splenic artery (splenic nerve) intraoperatively, but prior to kidney infection. The nerve fibres were blocked by loosely encircling the artery with a 5–0 silk suture soaked in 5 mg/ml bupivacaine (Accord Healthcare Limited). The bupivacaine-soaked sutures were left encircling the vessels from approximately 15 min prior to infection, throughout the experiment. In sham-blocked rats the vessels were encircled with sutures soaked in PBS. The bupivacaine solution was not administered systemically

### DRG extraction and cell culture

Primary mouse DRG cells were extracted from BALB/c mice (8–12 weeks of age) as previously described [46]. DRGs were collected from cervical to lumbar vertebral (C1-L6) levels under sterile conditions, and transferred to ice-cold HBSS media (GIBCO). DRGs were digested enzymatically first with 3 ml pre-warmed (37°C) papain (Worthington) solution (30U in HBSS media) supplemented with 0.1% saturated NaHCO_3_ (Merck) and 1 mg of L-cysteine (Sigma-Aldrich), and second with 4 mg/ml collagenase I (Worthington) and 4.5 mg/ml dispase II (Boehringer Mannheim) suspended in sterile pre-warmed HBSS. Incubations were performed at 37°C for 20 min each. Digested DRGs were then suspended in 1 ml of pre-warmed F12 media (GIBCO), supplemented with 10% Fetal Bovine Serum (GIBCO) and 1% penicillin/streptomycin (Invitrogen), and cells were mechanically separated with fire-polished glass Pasteur pipettes. The cell suspension was diluted with supplemented F12 media together with nerve growth factor (NGF 50ng/mL, Sigma-Aldrich) and the mitotic inhibitor 5-fluoro-deoxyuridine (FdUR 0.01mM, Sigma-Aldrich). Cells were seeded on coated coverslips (Laminin 0.02mg/mL, Sigma-Aldrich, and Poly-D-lysine 0.016 mg/mL, Becton Dickinson) in 24 well-plates (0.5mL/well) at a final concentration of 10^5^ cells/well. The viability of the cells was confirmed after 24 h, and after two days extending axons from the DRGs were confirmed.

On the day of the experiment cells were washed once with pre-warmed PBS before the addition of medium with or without stimuli. For bacterial stimulations 10^6^ CFU were used (MOI 1:10), and when indicated ATP (Sigma-Aldrich) at a concentration of 25 μM. For experiments investigating DRG CGRP release, capsaicin (10 μg/ml, Sigma-Aldrich) was used for positive controls. After 4 h stimulation supernatants were collected into Corning Costar Spin-X centrifuge filter tubes (Sigma-Aldrich) and centrifuged at 12000 x g for 1 minute, where after the filter was discarded. Supernatants were stored at -80°C until CGRP detection analysis.

### Renal epithelial cell culture and infection in the biomimetic flow model

The human epithelial cell line A498 (ATCC) was cultured in RPMI-1640 medium (Sigma-Aldrich) with 10% Fetal Bovine Serum and 1% GlutaMAX (ThermoFisher Scientific) or UltraCulture serum free media (Lonza) with 1% GlutaMAX, and incubated at 37°C and 5% CO_2_ for 24 h. One day prior to experiments, 2x10^4^-10^5^ A498 cells were seeded into a μ-slide VI^0.4^ (ibidi). On the day of experiment, one microchannel per condition was connected to a peristaltic pump. Cells were exposed to 75 μl/min of CO_2_ independent medium (CO2IM, ThermoFisher Scientific) supplemented with 10% FBS and 1% GlutaMAX or UltraCulture serum free media supplemented with 1% GlutaMAX. Cells stabilized under flow for 30 minutes before experiment start. The flow rate was calculated based on previous studies outlining the flow rate of primary filtrate in the S1 renal proximal tubule [47], as well as our experience micro-infusing at a rate which correlates to the observed physiological flow rate in the tubule (40 nl/min). The corresponding sheer stress in the microfluidic chambers was determined using the calculation provided by the manufacturer. Where indicated IFNγ (250 pg/ml) was added to the medium, and cells were pre-incubated with or without IFNγ for 1 h, where after bacteria were introduced to the channels. Bacteria were suspended in supplemented medium at a concentration of 10^8^ cells/ml and 0.5 ml was slowly introduced via an in-line LUER injection port (ibidi, t = 0 of the infection). Cells in the non-infected control microchannels were exposed only to supplemented media, without bacteria. Where indicated 250 pg/ml IFNγ was added to the flow through media of one channel after 1 h of infection. To screen host cells for apoptosis and necrosis, 5% Annexin V-Alexa Fluor 488 (ThermoFisher Scientific) in 1 x Annexin Buffer (ThermoFisher Scientific) was added to Ethidium Homodimer-1 (ThermoFisher Scientific) at a final concentration of 1.25 μM. 150 μl of this mix was injected in each microchannel through the in-line LUER injection port at different time points. The progression of the infection and the status of the host cells were captured with bright field and fluorescence microscopy (Nikon TS 100). 750 μl flow-through were collected at different time points from the outlet (10 min collection at 75 μl/min). CFT073 (HlyA+) and LT002 (HlyA-), the non-GFP expressing isogenic strains of LT004 (HlyA+) and LT005 (HlyA-), were used in some of these experiments to facilitate fluorescence imaging (**S1 Table** details bacterial strains).

### Hemolysis assay

50–75 μL of 2.5% human red blood cell (RBC) suspension in PBS were mixed with 50–75 μL (1:1 dilution) of filtered (0.2 um) samples collected during infection under flow conditions in triplicates in a 96-well plate. A positive control with 50–75 μL RBC + 50–75 μL 1% Sodium Dodecyl Sulphate (SDS, VWR) in CO2IM and a negative control with 50–75 μL RBC + 50–75 μL CO2IM, were also included in triplicates. The plate was incubated for 1 h at 37°C and centrifuged at 900 x g for 10 min at room temperature. Supernatants (80–100 μL) were transferred in a new plate and absorbance at measured at 405 nm (SynergyMix, BioTek). The average of the negative control absorbance values was subtracted from all other samples. The average of the absorbance values of all samples and the positive control was then calculated and constitutes n = 1 for each condition.

### Immunofluorescence and image processing

Kidneys or splenic tissue fixed in 4% paraformaldehyde (PFA, Sigma-Aldrich) were frozen in OCT (Fisher Scientific) and sectioned at 10 μm. Sections were stained with antibodies listed in **S2 Table** or treated with Hematoxylin and Eosin Y solutions (Sigma-Aldrich) for bright light microscopy. For immunofluorescent staining, samples were blocked with either 10% FBS or 3% Normal Donkey Serum and 0.1–0.3% TritonX100 (Sigma-Aldrich). Primary antibodies were diluted in blocking buffer, added to sections and incubated ON. After washing, secondary antibodies and dyes (phalloidin conjugated with FITC, Sigma-Aldrich, Hoechst 33258, Life Technologies, or DAPI, ThermoFisher Scientific) were added and incubated at RT for 2 h. In certain experiments (**Fig 2C and 2D**) the samples were thereafter subjected to increasing gradients (70%, 96% and 99.9%) EtOH and xylene. Sections were sealed with mounting media (Prolong Gold Antifade Mountant, ThermoFisher Scientific). Confocal imaging was performed using an Olympus FV1000 Confocal (BIC imaging facility, Karolinska Institutet), and bright light imaging was performed using an Olympus IX73 Inverted Microscope (BIC imaging facility, Karolinska Institutet). Images were processed using ImageJ. Final figures were prepared with Adobe Illustrator CS6.

### Quantitative real-time PCR

Splenic tissue from rats (~30 mg) was stored in RNA later RNA Stabilization Reagent (Qiagen) for 24 hours at 4°C, and thereafter stored at -80°C until extraction. Total RNA extraction was performed using RNEasy Mini kit (Qiagen) and a FastPrep-24 Homogenizer. The kit included an on-column DNA digestion step. cDNA was synthesized from 1 μg RNA using SuperScript III First Strand Synthesis Supermix kit (ThermoFisher Scientific). qPCR was performed using SYBR Green Master Mix (ThermoFisher Scientific) and a Quant Studio 5 PCR system. Primer sequences used are listed in **S3 Table**. In all experiments *Gapdh* was used as a reference gene.

### Detection assays

ELISA assays were performed on either homogenized rat tissue (~30 mg splenic or kidney tissue homogenized in PBS), plasma samples or cell culture supernatants/flow through using rat IFNγ ELISA development kit (Mabtech), rat IL-12/p40 ELISA kit (NordicBiosite), rat MPO ELISA kit (Aviva Systems Biology), human IL-8 ELISA development kit (Mabtech) and human IL-6 ELISA development kit (Mabtech). Assays of standards and samples were performed according to the manufacturer’s instructions. Cell culture supernatants were also analysed using rat-CGRP EIA kit (Bertin Pharma) and ATP Determination Kit (ThermoFisher Scientific) according to manufacturers’ instructions. For Luminex assays (R&D systems), either homogenized rat tissue or human cell culture flow through, was analysed according to the manufacturers’ instructions.

### Statistical analysis

Data were analysed with Prism 7 by Graph Pad. Results are expressed as medians or means ± SD or range as noted in figure legends. Mann-Whitney, Kruskal-Wallis, and one- or two-way ANOVA were used for analysis where appropriate (indicated in figure legends). Comparisons were made between all groups included in one figure, and if more than two groups were compared correction for multiple comparisons were made. P values of 0.05 or less were considered statistically significant and are represented in plots as *, #, or ##. Only significant differences are visualized in the figures.

## Supporting information

S1 Fig (**A**) CFU counts from blood at 4 h endpoint of animals infected with LT004 (HlyA+) and LT005 (HlyA-), or PBS infused, n = 5 in each group. (**B-C**) Hematoxylin and eosin staining of splenic tissues from rats after 4 h with (**B**) LT004-kidney infection and (**C**) PBS infusion. No red pulp (RP) congestion, no reduction in white pulp (WP) size, and no compression of central arteries (CA) is seen. Scale bars = 150 μm. Images are representative of n = 5. (**D**) At 4 h infection, the levels of IL12b/p40 in serum from animals infected with LT004 and LT005, or PBS infused are below the detection limit, n = 5. (**E-F**) Multiphoton microscopy of the microinfusion site at 3 h post infusion of (**E**) PBS or (**F**) LT004 (green). A 4 kDa TRITC-conjugated dextran (red) was co-infused to identify the infused tubule. Kidney autofluorescence is seen in green. Scale bars = 100 μm. Images are representative of n = 3–5. (**G**) CFU counts from blood at 4 h from sham-splenectomised (squares) or splenectomised (circles), and either infected (LT004, black symbols) or PBS-infused (PBS, unfilled symbols), n = 3–5 in each group. (**H**) E*x vivo* confocal imaging of splenic tissue from a sham-splenectomised animal 4h after PBS infusion. IFN-γ labelled with red, hoechst stain (blue) shows nucleated cells, scale bar = 50 μm. Image is representative of n = 3. Graphs show individual values and means (red bar). * = p<0,005, determined by Kruskal-Wallis and Dunn’s correction.
(TIF)

S2 Fig (**A**) Hemolytic activity, measured as arbitrary units (A.U.), in stationary cultures of CFT073 (HlyA+), LT002 (HlyA-), LT004 (HlyA+), and LT005 (HlyA-), as well as ARD371 (LT002 pBAD-HlyA) and ARD372 (LT005 pBAD-HlyA) in the presence of 0.2% arabinose. Graph shows data of one experiment representative of 3 individual experiments. (**B**) CGRP release from primary mouse DRG cultures stimulated with ARD372 (LT005 pBAD-HlyA), with capsaicin as positive control. Graph shows individual data points and mean (red bar), n = 4. * = p<0.005 calculated by one-way ANOVA and Turkey’s correction. (**C**) *Ex vivo* confocal imaging of kidney tissue 4 h after microinfusion of LT004 (HlyA+, green) shows bacteria (green) localized to the lumen (L) of a kidney tubule 4 h after microinfusion, and paracellular bacterial movement (arrow) toward the collagen IV-stained basement membrane (white). Scale bar = 25 μm. Image is representative of n = 5. (**D**) CGRP release from primary mouse DRG cultures stimulated with ATP, with capsaicin as positive control. Graph shows individual data points and mean (red bar), n = 3. * = p<0.005 calculated by one-way ANOVA and Turkey’s correction. (**E**) Bacterial cultures of CFT073 (HlyA+), LT002 (HlyA-), and ARD371 (LT002 pBAD-HlyA) have no significant eATP release at any timepoint up to 4 h, calculated by two-way ANOVA and Turkey’s correction. Media without bacteria is shown as control. Graph shows means ± SD, n = 3. (**F-G**) Repeats (in total n = 3) of experiment from Fig 3C, showing hemolytic activity in flow-through media from A498 cells infected with CFT073 (HlyA+, black circles), LT002 (HlyA-, black triangles) or uninfected (unfilled circles). (**H-J**) Hemolytic activity in flow-through media from A498 cells infected with ARD371 (LT002 pBAD-HlyA, black diamonds) compared to CFT073 (HlyA+, black circles), LT002 (HlyA-, black triangles) or uninfected (unfilled circles). (**K**) Bright field microscopy of A498 cells cultured under flow, infected with CFT073 (HlyA+, bottom panels), LT002 (HlyA-, middle panels) or uninfected (top panels). (**L**) Fluorescence microscopy of infected and uninfected A498 cells at 4 h. Cells stained for apoptosis (Annexin V, green) or necrosis (EthD-1, red). Images in (**K**) and (**L**) are representative of n = 3, and scale bar = 50 μm. Arrows indicate flow direction. (**M**) *Ex vivo* confocal imaging of kidney tissue 4 h after microinfusion of LT005 (HlyA-, green) shows similar pathophysiological changes as LT004 (HlyA+), with bacteria (green) localized to the lumen (L) and paracellular bacterial movement (arrow) toward the collagen IV-stained basement membrane (white). Scale bar = 25 μm. Image is representative of n = 5. (**N**) E*x vivo* confocal imaging of splenic tissue from a sham-splenectomised animal 4h after microinfusion of LT004. O6-antigen (LPS, red) was not found. Hoechst stain (blue) shows nucleated cells. Scale bar = 50 μm. Image is representative of n = 5.
(TIF)

S3 Fig (**A-G**) Luminex analysis of cytokine profiles and (**H**) MPO levels measured by ELISA in kidney biopsies taken from rats who underwent sham-splenectomy (circles) or splenectomy (squares), and were either infected (LT004, black symbols) or PBS infused (PBS, unfilled symbols). Individual data points and median values (red bars) are plotted, n = 3–5 in each group. No significant difference between the groups could be determined by Kruskal-Wallis with Dunn’s correction. ND = not detected.
(TIF)

S4 Fig (**A-H**) Luminex analysis of flow through media of renal epithelial A498 cells either infected with LT004 (black circles), or uninfected (unfilled circles), at designated time points. Graphs show means ± SD, n = 4. * = p<0.05 (p-values are noted in the figures where appropriate), determined by two-way ANOVA and Bonferroni’s correction. ND = not detected. (**I**) CFU counts of LT004 bacteria cultured in serum free media with increasing concentrations of IFNγ for 4 h. No statistical difference was found by one-way ANOVA and Turkey’s correction. Only serum free media, without any bacteria is shown for reference. (**J**) Bright field microscopy of A498 cells cultured under flow, either uninfected, infected with LT004 or infected with LT004 in the presence of 250 pg/ml IFNγ, at 4 h. Arrow indicates flow direction. Images are representative of n = 3, and scale bar = 50 μm.
(TIF)

S1 Table Bacterial strains and primers for genetic complementation. (DOCX)

S2 Table Antibodies used for immunofluorescent staining. (DOCX)

S3 Table Primers used for qPCR. (DOCX)

S1 Video Time-lapse recording of renal epithelial A498 cells infected with LT002 (HlyA-). Video is captured at 5 h of infection, using a recording speed of 10 fps. Playback speed = 10 fps, scale bar = 100 μm, arrow = flow direction, time = hh:mm:ss. One representative experiment of 3 is shown.
(AVI)

S2 Video Time-lapse recording of renal epithelial A498 cells infected with CFT073 (HlyA+). Video is captured at 4–5 h of infection, using a recording speed of 2 frames per min. Playback speed = 5 fps, scale bar = 100 μm, arrow = flow direction, time = hh:mm. One representative experiment of 3 is shown.
(AVI)
