# Supplementary figures and images for: UPEC kidney infection triggers neuro-immune communication leading to modulation of local renal inflammation by splenic IFNγ
Source: PLoS Pathog. 2021 May 20;17(5):e1009553. doi: 10.1371/journal.ppat.1009553 (PMC8136731; doi:10.1371/journal.ppat.1009553)

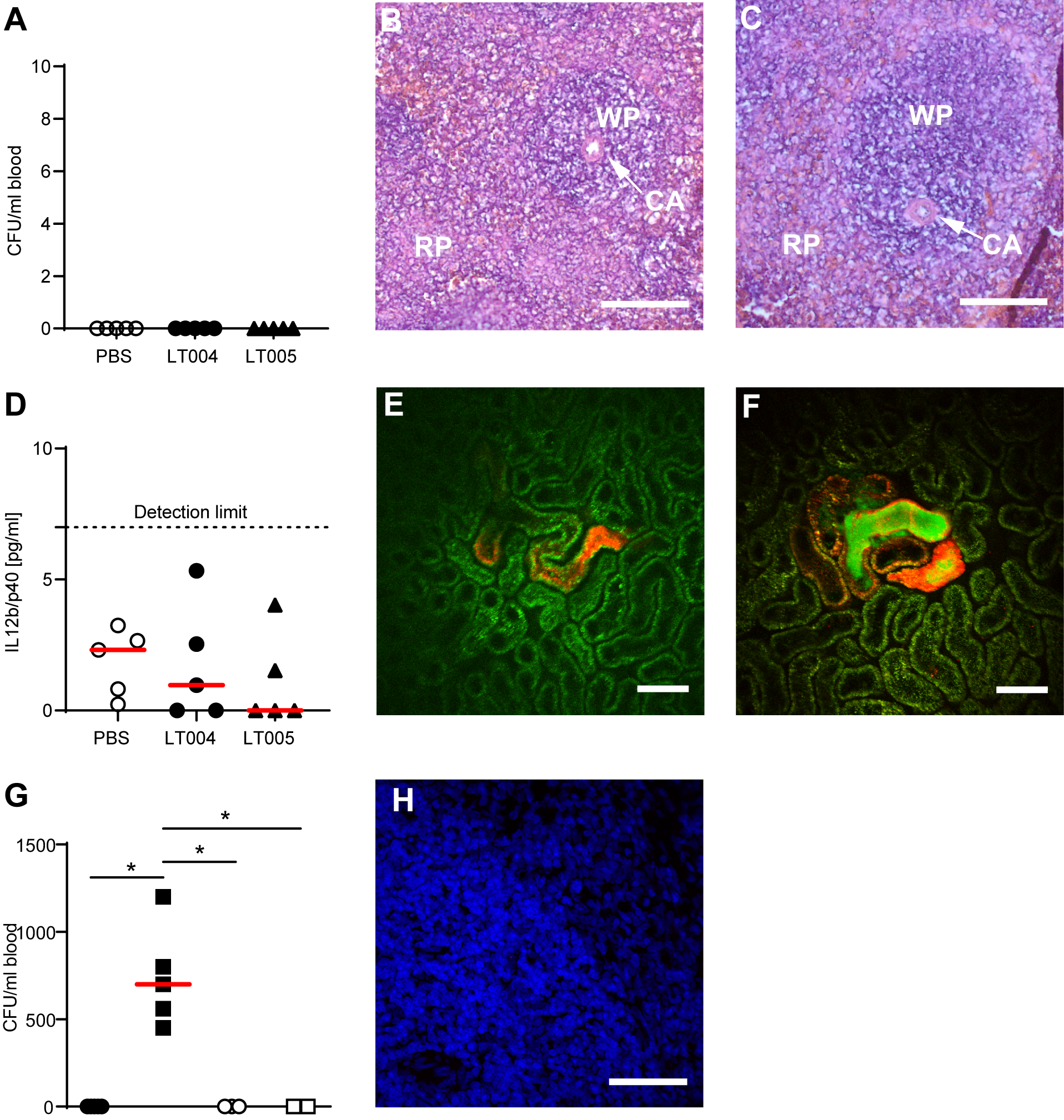

Supplement: S1 Fig — (A) CFU counts from blood at 4 h endpoint of animals infected with LT004 (HlyA+) and LT005 (HlyA-), or PBS infused, n = 5 in each group. (B-C) Hematoxylin and eosin staining of splenic tissues from rats after 4 h with (B) LT004-kidney infection and (C) PBS infusion. No red pulp (RP) congestion, no reduction in white pulp (WP) size, and no compression of central arteries (CA) is seen. Scale bars = 150 μm. Images are representative of n = 5. (D) At 4 h infection, the levels of IL12b/p40 in serum from animals infected with LT004 and LT005, or PBS infused are below the detection limit, n = 5. (E-F) Multiphoton microscopy of the microinfusion site at 3 h post infusion of (E) PBS or (F) LT004 (green). A 4 kDa TRITC-conjugated dextran (red) was co-infused to identify the infused tubule. Kidney autofluorescence is seen in green. Scale bars = 100 μm. Images are representative of n = 3–5. (G) CFU counts from blood at 4 h from sham-splenectomised (squares) or splenectomised (circles), and either infected (LT004, black symbols) or PBS-infused (PBS, unfilled symbols), n = 3–5 in each group. (H) Ex vivo confocal imaging of splenic tissue from a sham-splenectomised animal 4h after PBS infusion. IFN-γ labelled with red, hoechst stain (blue) shows nucleated cells, scale bar = 50 μm. Image is representative of n = 3. Graphs show individual values and means (red bar). * = p<0,005, determined by Kruskal-Wallis and Dunn’s correction. (TIF) [file ppat.1009553.s001.tif]

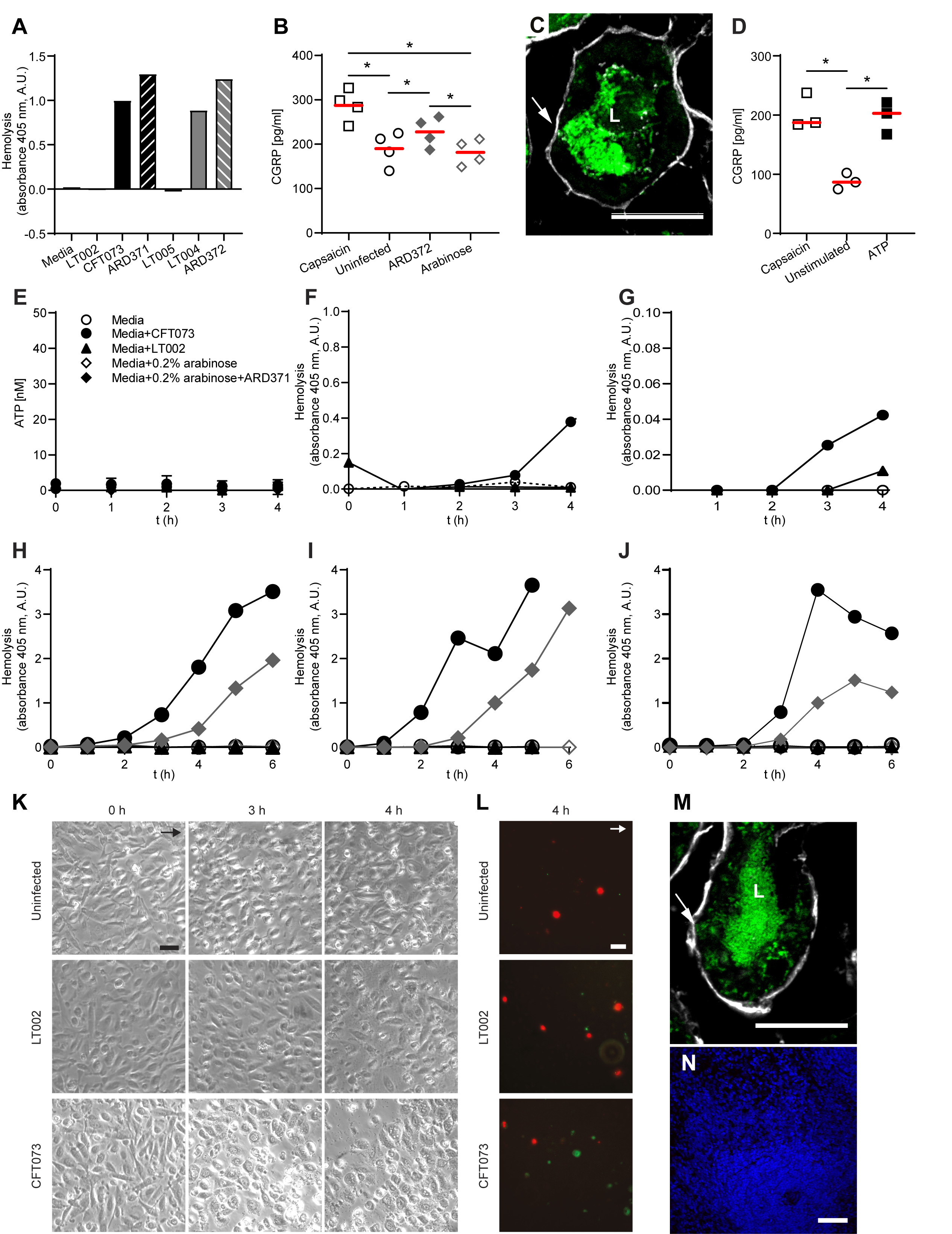

Supplement: S2 Fig — (A) Hemolytic activity, measured as arbitrary units (A.U.), in stationary cultures of CFT073 (HlyA+), LT002 (HlyA-), LT004 (HlyA+), and LT005 (HlyA-), as well as ARD371 (LT002 pBAD-HlyA) and ARD372 (LT005 pBAD-HlyA) in the presence of 0.2% arabinose. Graph shows data of one experiment representative of 3 individual experiments. (B) CGRP release from primary mouse DRG cultures stimulated with ARD372 (LT005 pBAD-HlyA), with capsaicin as positive control. Graph shows individual data points and mean (red bar), n = 4. * = p<0.005 calculated by one-way ANOVA and Turkey’s correction. (C) Ex vivo confocal imaging of kidney tissue 4 h after microinfusion of LT004 (HlyA+, green) shows bacteria (green) localized to the lumen (L) of a kidney tubule 4 h after microinfusion, and paracellular bacterial movement (arrow) toward the collagen IV-stained basement membrane (white). Scale bar = 25 μm. Image is representative of n = 5. (D) CGRP release from primary mouse DRG cultures stimulated with ATP, with capsaicin as positive control. Graph shows individual data points and mean (red bar), n = 3. * = p<0.005 calculated by one-way ANOVA and Turkey’s correction. (E) Bacterial cultures of CFT073 (HlyA+), LT002 (HlyA-), and ARD371 (LT002 pBAD-HlyA) have no significant eATP release at any timepoint up to 4 h, calculated by two-way ANOVA and Turkey’s correction. Media without bacteria is shown as control. Graph shows means ± SD, n = 3. (F-G) Repeats (in total n = 3) of experiment from Fig 3C, showing hemolytic activity in flow-through media from A498 cells infected with CFT073 (HlyA+, black circles), LT002 (HlyA-, black triangles) or uninfected (unfilled circles). (H-J) Hemolytic activity in flow-through media from A498 cells infected with ARD371 (LT002 pBAD-HlyA, black diamonds) compared to CFT073 (HlyA+, black circles), LT002 (HlyA-, black triangles) or uninfected (unfilled circles). (K) Bright field microscopy of A498 cells cultured under flow, infected with CFT073 (HlyA+, bottom panels) [file ppat.1009553.s002.tif]

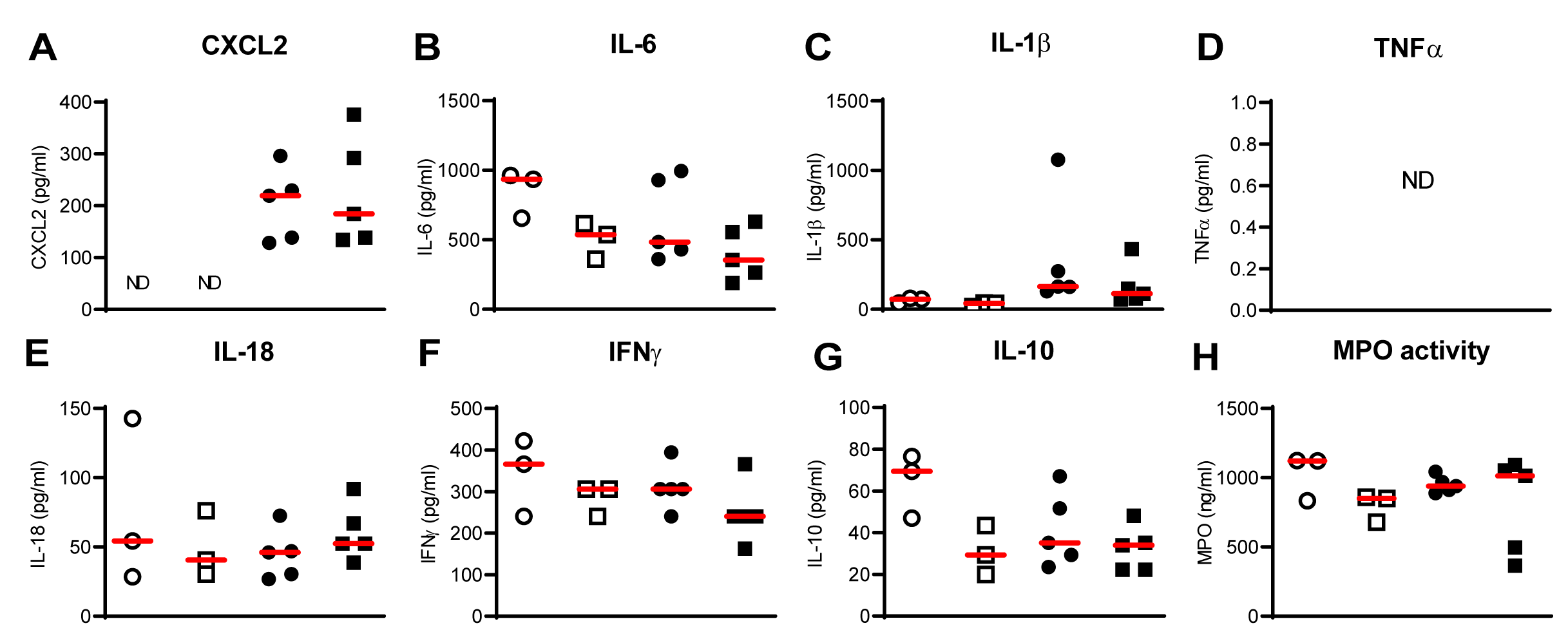

Supplement: S3 Fig — (A-G) Luminex analysis of cytokine profiles and (H) MPO levels measured by ELISA in kidney biopsies taken from rats who underwent sham-splenectomy (circles) or splenectomy (squares), and were either infected (LT004, black symbols) or PBS infused (PBS, unfilled symbols). Individual data points and median values (red bars) are plotted, n = 3–5 in each group. No significant difference between the groups could be determined by Kruskal-Wallis with Dunn’s correction. ND = not detected. (TIF) [file ppat.1009553.s003.tif]

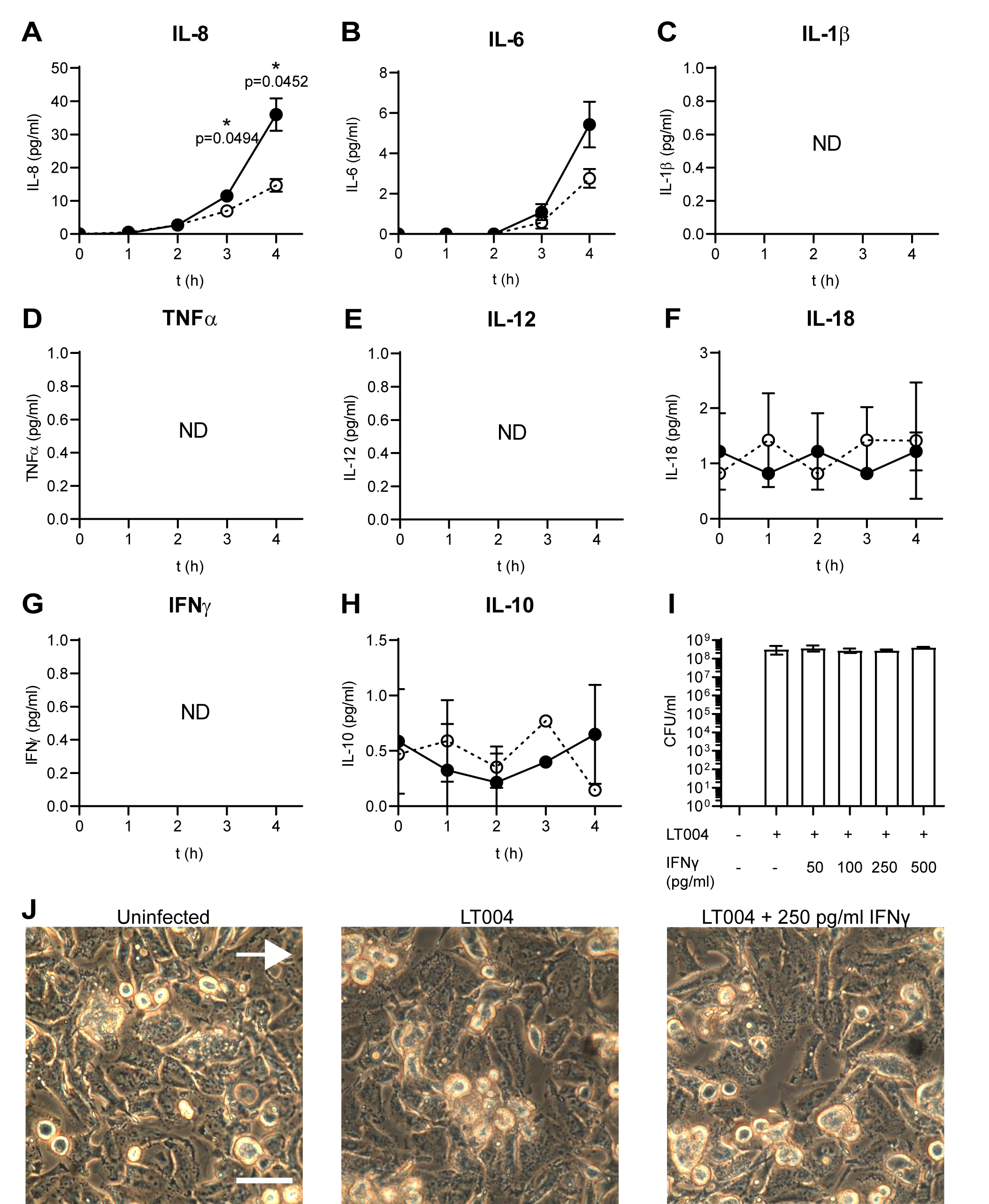

Supplement: S4 Fig — (A-H) Luminex analysis of flow through media of renal epithelial A498 cells either infected with LT004 (black circles), or uninfected (unfilled circles), at designated time points. Graphs show means ± SD, n = 4. * = p<0.05 (p-values are noted in the figures where appropriate), determined by two-way ANOVA and Bonferroni’s correction. ND = not detected. (I) CFU counts of LT004 bacteria cultured in serum free media with increasing concentrations of IFNγ for 4 h. No statistical difference was found by one-way ANOVA and Turkey’s correction. Only serum free media, without any bacteria is shown for reference. (J) Bright field microscopy of A498 cells cultured under flow, either uninfected, infected with LT004 or infected with LT004 in the presence of 250 pg/ml IFNγ, at 4 h. Arrow indicates flow direction. Images are representative of n = 3, and scale bar = 50 μm. (TIF) [file ppat.1009553.s004.tif]
