# Supplementary material for: UPEC kidney infection triggers neuro-immune communication leading to modulation of local renal inflammation by splenic IFNγ
Source: PLoS Pathog. 2021 May 20;17(5):e1009553. doi: 10.1371/journal.ppat.1009553 (PMC8136731; doi:10.1371/journal.ppat.1009553)
Supplement: S1 Table — (DOCX) [file ppat.1009553.s005.docx]

| **Strain name** | **Description** | **LPS** | ***HlyA+*** | ***GPF+*** | **Source/Reference** |
| --- | --- | --- | --- | --- | --- |
| CFT073 | 06:K2:H1 | 06 | Yes | No | Mobley et al. (1990); Welch et al. (2002) |
| LT002 | CFT073 hlyA::km^R^ | 06 | No | No | Månsson et al. (2006) |
| LT004 | CFT073 cobS::Φ(PLtetO-1-gfp+), cm^R^ | 06 | Yes | Yes | Månsson et al. (2006) |
| LT005 | LT002 cobS::Φ(PLtetO-1-gfp+), cm^R^, km^R^ | 06 | No | Yes | Månsson et al. (2006) |
| ARD371 | LT002 with pBAD-HlyA, amp^R^ | 06 | Yes | No | This work |
| ARD372 | LT005 with pBAD-HlyA, amp^R^ | 06 | Yes | Yes | This work |
| **Primer name** | **Forward 5’ -> 3’** | **Reverse 5’-> 3’** | | | |
| SacI_hlyA_FW | ATAGAGCTCATGCCAACAATAACCACTGCAC |  | | | |
| BstBI_hlyA_RV |  | ACATTCGAATTATGCTGATGCTGTCAAAGTTATTG | | | |
