# Supplementary material for: UPEC kidney infection triggers neuro-immune communication leading to modulation of local renal inflammation by splenic IFNγ
Source: PLoS Pathog. 2021 May 20;17(5):e1009553. doi: 10.1371/journal.ppat.1009553 (PMC8136731; doi:10.1371/journal.ppat.1009553)
Supplement: S2 Table — (DOCX) [file ppat.1009553.s006.docx]

| **Antibody** | **Source** | **Identifier** |
| --- | --- | --- |
| Rb α-O6 antigen | Statens Serologiska Institut, Denmark |  |
| Rb α-Collagen IV | Abcam | ab6586-100 |
| Ms α-Rt β3 tubulin | Novus | nb600-1018 |
| Rb α-PGP9.5 | Cedarlene | CL7756AP |
| Gt α-Rt TrkA | R&D Systems | AF1056 |
| Gt α-Rt IFNγ | R&D Systems | AF-585-NA |
| Dk α-Rb Cy2 | Jackson Immuno Research | 711-225-152 |
| Gt α-Rb Cy3 | Jackson Immuno Research | 111-165-003 |
| Dk α-Gt Cy3 | Jackson Immuno Research | 705-165-147 |
| Dk α-Ms Cy3 | Jackson Immuno Research | 715-165-151 |
| Dk α-Rb Cy5 | Jackson Immuno Research | 711-175-152 |
| Dk α-Ms Cy5 | Jackson Immuno Research | 715-175-151 |
| Dk α-Gt NL557 | R&D Systems | NL001 |
