# Supplementary material for: UPEC kidney infection triggers neuro-immune communication leading to modulation of local renal inflammation by splenic IFNγ
Source: PLoS Pathog. 2021 May 20;17(5):e1009553. doi: 10.1371/journal.ppat.1009553 (PMC8136731; doi:10.1371/journal.ppat.1009553)
Supplement: S3 Table — (DOCX) [file ppat.1009553.s007.docx]

| **Rat gene** | **Forward 5’ -> 3’** | **Reverse 5’-> 3’** |
| --- | --- | --- |
| *Gapdh* | GTATGATGACATCAAGAAGGTGG | CATTGTCATACCAGGAAATGAG |
| *Ifng* | GAGCCAGATTATCTCTTTCTACC | GTTGTTCACCTCGAACTTGG |
| *Il12a* | GCTAATGCAGTCTCTGAATCAC | GCTCAGATAGTTCATCACCCT |
| *Il12b* | GAGTGTAACCAGAAAGGTGC | TGCATGATGAATTGTAGTAGCG |
| *Il17a* | GTACTCATCCCTCAAAGTTCAG | CACAGAAGGATATCTATCAGGGTC |
| *Il23a* | TGGGACAAATGGATCTACTAAGAG | AGTAGGGAGGTATGAAGCTG |
| *Cxcl1* | GCGGAGAGATGAGAGTCTGG | AGGCATTGTGCCCTACAAAC |
